# Electrical Strength of Natural Esters Doped by Iron Nanopowder in a Hydrophobic Carbon Shell

**DOI:** 10.3390/ma13081956

**Published:** 2020-04-22

**Authors:** Łukasz Nagi, Aleksandra Płużek

**Affiliations:** Faculty of Electrical Engineering, Automatic Control and Informatics, Opole University of Technology, Proszkowska 76, 45-758 Opole, Poland; aleksandra.pluzek@student.po.edu.pl

**Keywords:** iron nanoparticles, Midel 1204, liquid insulator, breakdown voltage, electrical strength

## Abstract

The paper presents the results of measurements of electrical strength of Midel 1204 natural ester doped with iron nanopowder in a hydrophobic carbon shell. The research was conducted for different concentrations of the dopant. The samples were prepared in the High Voltage Technique Laboratory. After mixing, they were tightly closed, and the first measurements were taken after 5 weeks of dissolution of the dopant in liquid. The tests were repeated after another 2 weeks and 3 weeks of dissolution of nanoparticles. An increase in both mean and maximum breakdown voltage was shown for the tested liquid mixtures. The concentration for which the value of electrical strength begins to decrease was indicated. It was also shown that a longer time of dissolution of nanoparticles causes an increase in the electric strength value for the tested samples.

## 1. Introduction

The aim of the conducted research was to check how the electrical strength of the natural ester Midel 1204 will be determined by the addition of iron nanopowder with hydrophobic carbon shells in various concentrations. In power systems where oils or esters are both electrical insulators and coolants, the complete replacement of used oil is quite expensive, and the aged transformer oil is environmentally hazardous. For this reason, intensive research is being carried out on substitutes and on modification of existing substances increasing their service life and resistance to phenomena affecting them.

The main feature of nanoparticles is that adding them to different substances can change selected properties of the materials being doped or create completely new ones. Nanoparticle doping is used in many fields of science and industry. Medical research carried out in recent years has shown that, e.g., gold nanoparticles can be useful as a contrasting substance, using X-rays, which offers more advantages compared to standard iodine-based products [1]. Other studies [2] show the potential of zinc oxide nanoparticles for use in photovoltaics to improve the performance of photovoltaic cells. In turn, one of the more recent studies in the field of industry [3] shows that the nanoparticles of copper and cobalt admixed to PVDF-HFP film can be used in electronics, and the magnetic nanoparticles described in [4] can be potentially used in the oil industry.

The recent interest in the development of nanomaterials has led researchers to the study of their electrical properties and the applications that they may have as insulating materials. The articles [5,6,7,8] focus on research in electro-insulating liquids. In [5], the authors described the effect of activated bentonite on aged transformer oil, where the electrical, physical, chemical, and thermal properties were significantly improved. The studies presented in [6,7] show the analysis of thermal properties of mineral oil, synthetic ester, and natural ester after admixture of fullerene C_60_ nanoparticles and titanium oxide TiO_2_, which also showed positive effects on oils. There are also many studies on the use of iron oxides in electro-insulating liquids in the literature. In [8], two types of modified natural esters containing colloidal nanoparticles of iron oxide and silica, which had a positive effect on the liquids, causing an increase in the sample breakthrough voltage, were studied. Research on fluids used in transformers as insulators and cooling fluids is carried out not only for electrical strength, but also for thermal conductivity, lifetime, and many other physicochemical parameters. Authors obtained a 61% and 50% increase in the breakdown voltage for nanofluids with iron oxide, which caused interest in other iron compounds added to transformer oils. Iron itself is a good electrical conductor, which is not the preferred oil additive. Iron oxides, on the other hand, have completely different properties, which is why it was decided to study the effect of the material in the carbon shell on the electrical strength of the liquid. The hydrophobic shell has the additional advantage in that it can increase the oil’s resistance to moisture and, at the same time, makes the iron not a contaminant. In [9], research on the lightning impulse breakdown voltage of mineral oil and several Fe_3_O_4_-based nanofluids is presented. The authors of [10] demonstrated that iron oxide after mixing with fresh ester oil can improve electro-insulating properties of liquids.

Mineral oils and esters used in transformers are aged depending on the conditions in which they work. This is influenced by both transformer loads and damage. Defects occurring in such systems are both an effect and a cause of aging of electro-insulating liquids. The main problem is the partial discharge (PD) occurring in transformers. Diagnostics of power equipment and networks is based on the examination of phenomena accompanying PD in different configurations. PD is generated in laboratory conditions, e.g., long-term AC voltage [11], and measured using various methods. Acoustic signals from the range of ultrasound or infrasound are recorded using piezoelectric sensors [12,13] in various electro-insulating liquids [14]. The most commonly used method is the registration of apparent charge based on the standard [15]. The methods recording electromagnetic radiation generated during PD are becoming more and more popular. The oldest of them is the recording of radiation in the UV range with a UV camera. A development of this method is the use of a spectrophotometer to record the optical spectrum in a much wider range from UV through VIS to near IR. The measurement systems and methodology are presented, e.g., in [16,17]. The electromagnetic radiation coming from PD is also registered in the Ultra high frequency (UHF) range [18] and high ionizing energies [19,20]. Not only measuring devices, systems, and methods, but also all kinds of descriptors have been developed to assess the damage in both liquid and gas and solid insulators. However, new isolating liquids may have different physicochemical properties, and thus, the signals emitted by PD may differ from their basic forms. Research on insulating fluids doped with nanoparticles is a new branch of research. However, before new diagnostic methods are developed or modified, the basic properties of new nanofluids should be studied.

The hydrophobicity of insulation materials is also very important. Resistance to moisture, and especially to its absorption, has a significant impact on both electrical strength and the effects of aging the insulation. If the dielectric material becomes moist, its electrical strength decreases. Additionally, the lifetime of such material decreases drastically. As shown in [21], the reduction of hydrophobicity of insulators results in their faster degradation. Water contained in the esters is generally present in the dissolved and undissolved form. Despite the fact that esters are highly hydrophobic, temperature rise has a large effect on the oil water solubility.

Since various forms of carbon structures are hydrophilic, e.g., fullerenes [22,23], which are also used in scientific research as an admixture to transformer mineral oils, and the humidity of the insulating liquid is not appropriate, the authors decided to use molecules with a hydrophobic carbon shell. The studies presented in this article analyzed Midel 1204 ester doped with iron-based nanopowder in a hydrophobic carbon shell.

## 2. Materials and Methods

**Figure 1:**
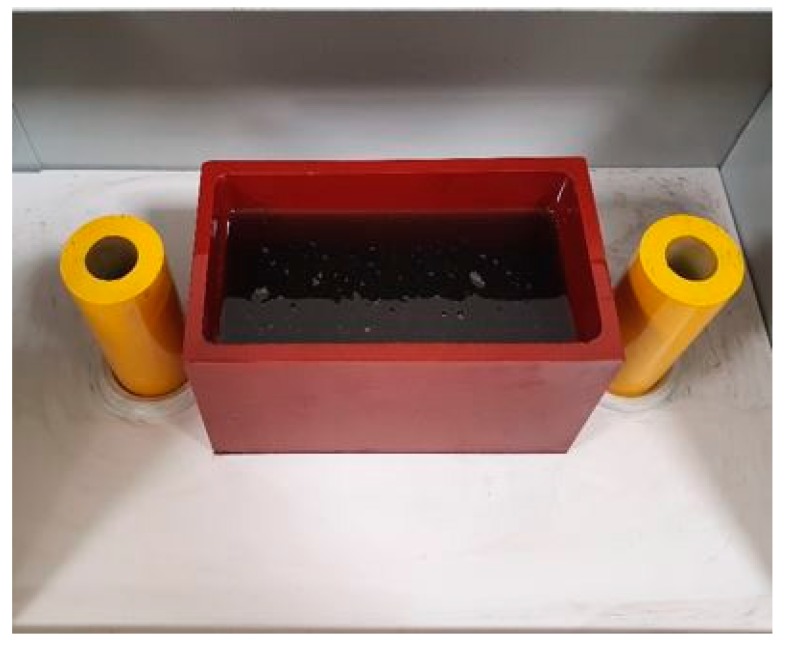
Oil tank with tested liquid.

**Figure 2:**
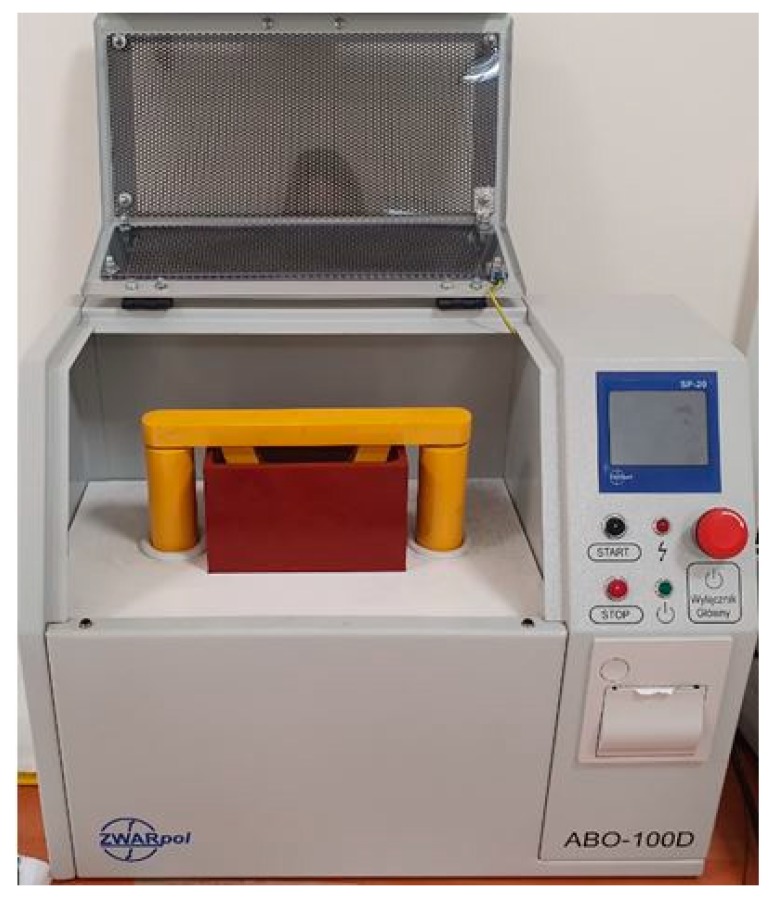
Breakdown voltage tester.

**Figure 3:**
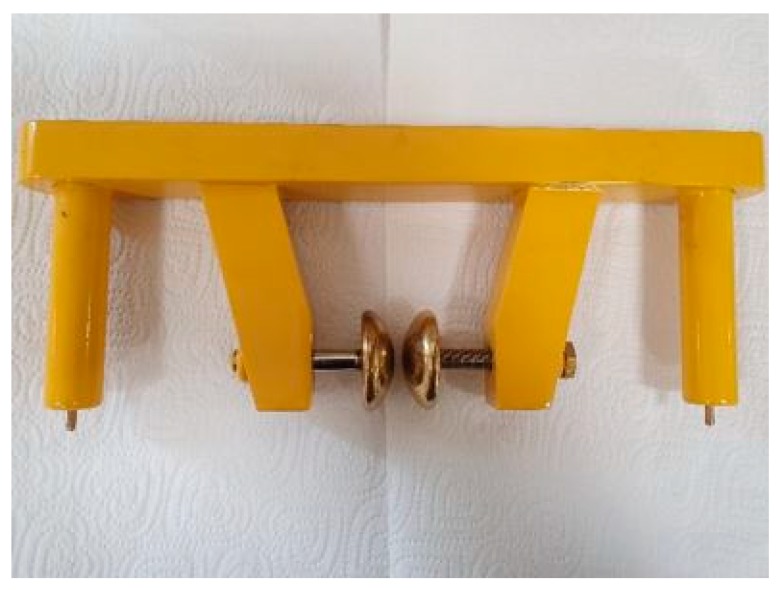
Spherical electrodes used for testing.

The aim of the study was to check how selected electro-insulating properties of the Midel 1204 ester will be affected by the addition of iron nanopowder with hydrophobic carbon shell in various concentrations. Midel 1204 with a density of 0.92 g/cm^3^ was used as the base liquid. The volume of liquid of each sample was 750 mL. The nanoparticles were weighed using electronic scales and their concentration in oil was calculated. The concentrations of nanoparticles in the samples reached 0.0007%, 0.0017%, 0.0049%, and 0.0188%. Used additive is the commercial product. The chemical composition of nanopowder was >83% Fe and <14% C and was determined by supplier (PlasmaChem GmbH, Berlin, Germany). The next step was to measure the breakdown voltage for pure Midel 1204 ester. The breakdown voltage after dissolution of nanoparticles in the liquid was also measured according to the “Insulating liquids—Determination of the breakdown voltage at power frequency—Test method,” Int. Stand. IEC 60156 [15]. The nanofluid was filled into the tank shown in Figure 1, which was then placed in the liquid’s electrical strength testing device. To perform the tests, ZWARpol’s oil puncture voltage tester, shown in Figure 2, was used. Its power is 220 VA, the maximum secondary voltage is 2 × 50 kV, and the test voltage is 2 × 60 kV. The next step was to insert into the nanofluid spherical electrodes shown in Figure 3, which were invariably 1 cm distance from each other. One of them was grounded and a voltage was applied to the other, which was changed continuously until the breakdown occurred. The speed of the voltage increment was 2 kV/s.

To test the effect of the compound on the Midel 1204 ester properties (M&I Materials Ltd, Hibernia Way, Trafford Park, Manchester M32 0ZD, United Kingdom), the samples were mixed and the time after which the tests on the mixtures were performed was 5 weeks. After that time, there was no visible sediment, which indicated a better dissolution of the additive in oil. After doping, the nanoparticle fell to the bottom of the glassware without modifying the colour of the base liquid initially. After 5 weeks, the samples became black, depending on the concentration contained. The dissolution process was supported by 5 min manual mixing with a glass rod before each measurement. Breakdown voltage tests for each nanofluid in 5, 7, and 10 weeks were performed six times. For each nanofluid, 24 measurement tests were performed and for pure Midel 1204, 6 in a total of 78 measurement tests (after IEC 60156 standard). This methodology was previously used in studies with C_60_ fullerenes and described in [23]. The article describes the properties of aged mineral oil with admixture of fullerenes. The samples were stored while dissolving the admixtures in such a way as to prevent their moistening. Measurements for each nanoliquid were taken from about 15 to 17 min, including the time of liquid mixing between electric strength tests, which in total took 2 min. The measurement results were analyzed in MATLAB (ver. 2019b, MathWorks, Natick, MA, USA). The samples were tested again after the next 2 weeks and next 3 weeks.

## 3. Results and Discussion

**Figure 4:**
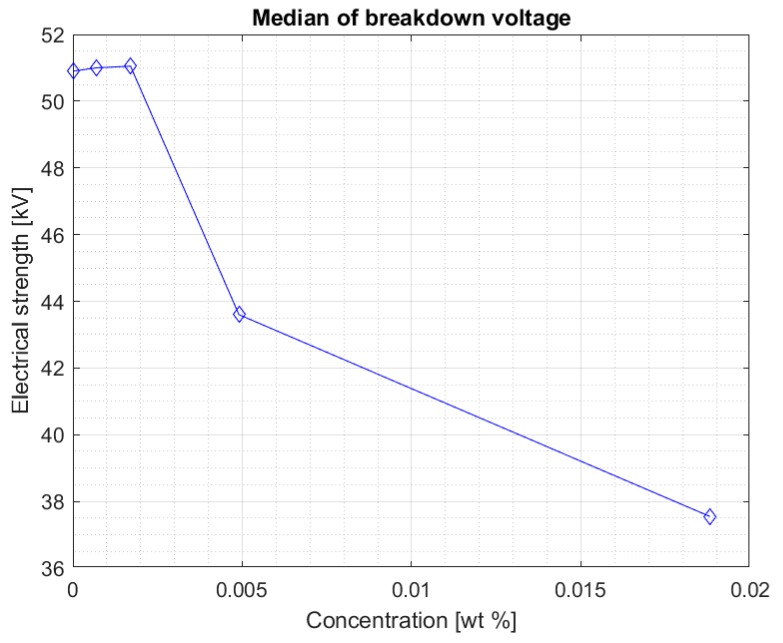
Median of measurement results for each nanofluid five weeks after mixing.

The aim of the study was to investigate the effect of iron nanoparticles in the carbon shell on the electrical strength of the Midel 1204 ester. Figure 4 shows the median of the results obtained for each set of measurements of the sample breakdown voltage after doping and mixing, depending on the concentration of iron nanoparticles in the hydrophobic carbon shell. It suggests that a certain concentration of the doping does not negatively affect the liquid. The value of the breakdown voltage even increases slightly.

**Figure 5:**
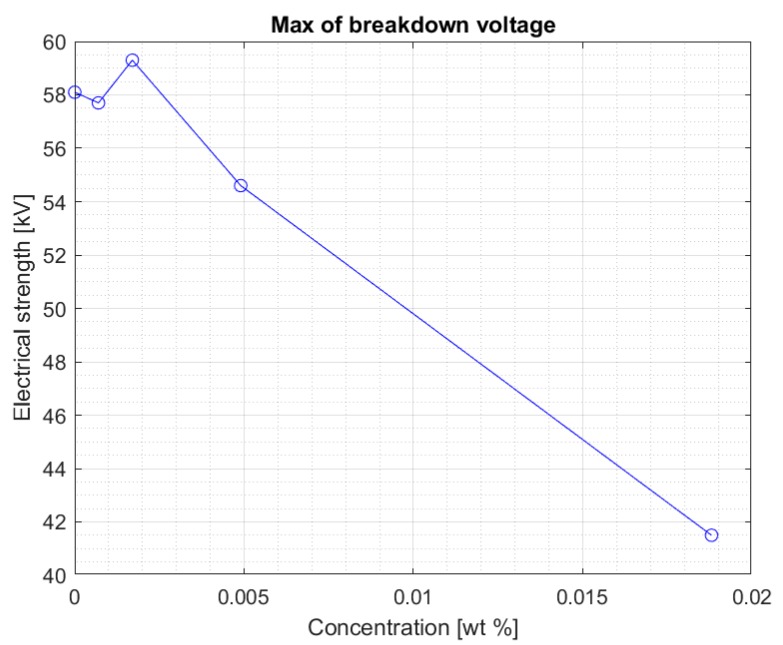
Maximum breakdown voltage values achieved for different nanoparticle concentration in the doped ester five weeks after mixing.

**Figure 6:**
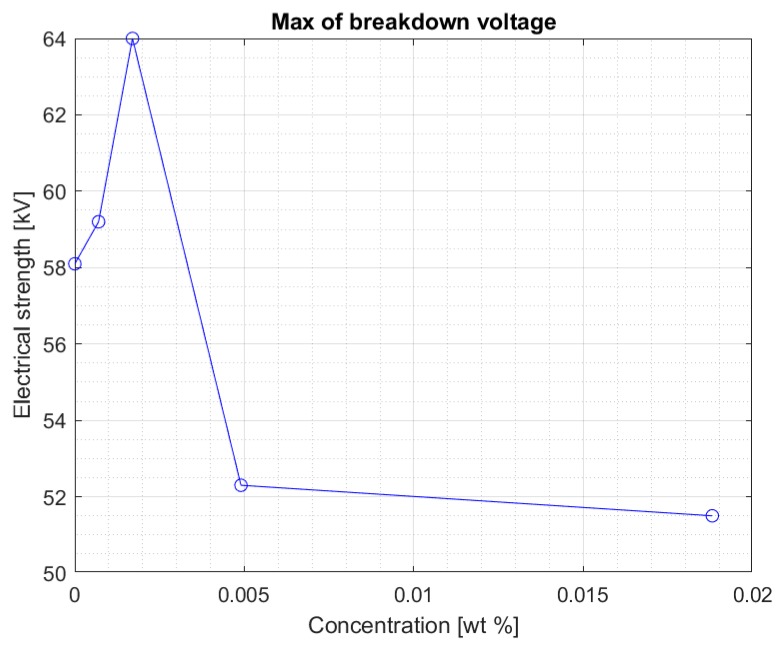
Maximum breakdown voltage values achieved for different nanoparticle concentration in the doped ester seven weeks after mixing.

**Figure 7:**
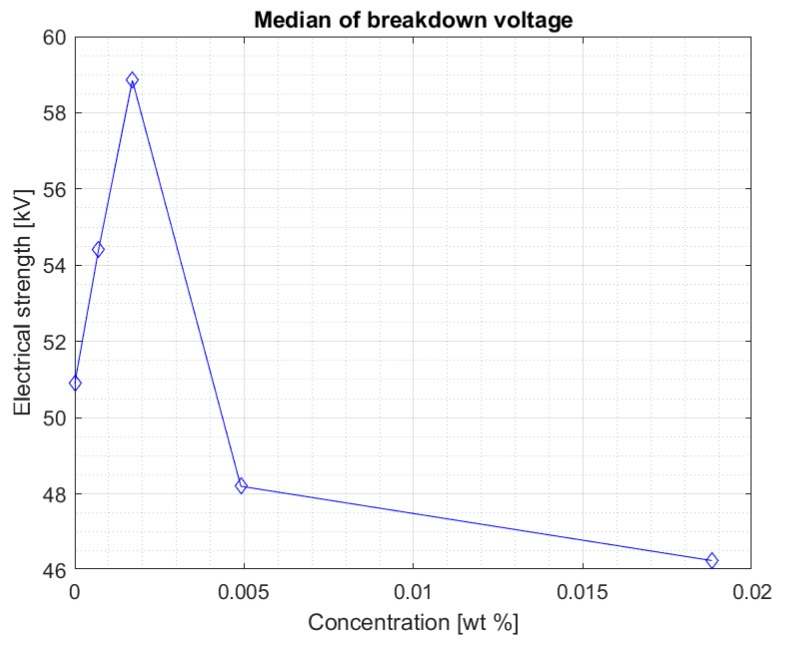
Median of measurement results for each nanofluid seven weeks after mixing.

The values are stable within the standard deviation. Above the concentration of 0.0017% of the nanoparticle in the liquid, a clear decrease in the electrical strength of the nanofluid is visible. This is confirmed by another graph (Figure 5) showing the maximum breakdown voltage values obtained for nanofluids with four tested concentrations of iron nanoparticles. Figure 6 and Figure 7 show the maximum voltage and the median of the breakdown voltage values for the liquids tested after 2 weeks from the first measurement. A significant increase in the maximum obtained breakdown voltage in the system can be observed for some concentrations. It can be assumed with some approximation that this is related to the percolation threshold. As the concentration of the admixture increases, the time needed for its complete dissolution increases. At the same time, too high of an admixture concentration may not dissolve completely in Midel 1204 even after 10 weeks. The samples were mixed with a stirrer to support the process of dissolving the admixture in the entire sample volume. It was noted, however, that at higher concentrations during measurements, undissolved nanoparticles (invisible before the test) were concentrated in the electrode area after each strength test. Considering that one of the elements in the admixture was iron, the electric field during the voltage increase between the electrodes could concentrate the still undissolved nanoparticles. After each test, the sample was mixed to average the concentration of the admixture in the sample. However, the formation of such aggregations during the measurement is likely to reduce the electrical strength of the liquid as suggested in article [24,25] with research in solid dielectrics doped with nanoparticles. In this situation, the nanoparticles tend to be in contact with each other, thus forming a continuous assembly. This can result in important changes of the physical properties which no longer increase the electrical strength.

Above a certain value, the results suggest that too high of a concentration of nanoparticles in fresh Midel 1204 ester causes a decrease in its electrical strength (Figure 6). Figure 7 shows the median for measurements in the Midel 1204 ester after 7 weeks from doping. The significant difference in results between the measurements after 5 weeks and 7 weeks after doping suggests that, as with fullerenes, the dissolution time of the additives is important and positively affects the results. An increase in electrical strength can be seen for samples with a nanoparticle concentration of 0.0007% and 0.0017% compared to the results obtained during the first measurement, the median of which can be seen in Figure 4. Comparison of both graphs also suggests a slight increase in strength for the remaining nanofluids, but still, above the nanoparticle concentration of 0.0017%, a significant decrease in electrical strength of the sample compared to pure Midel 1204 ester can be seen.

**Figure 8:**
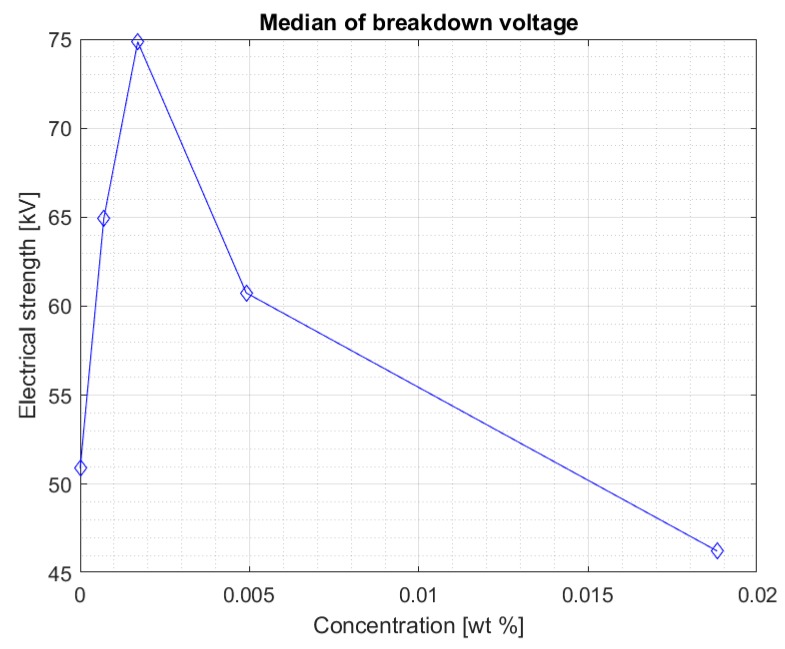
Median of measurement results for each nanofluid after ten weeks mixing.

**Figure 9:**
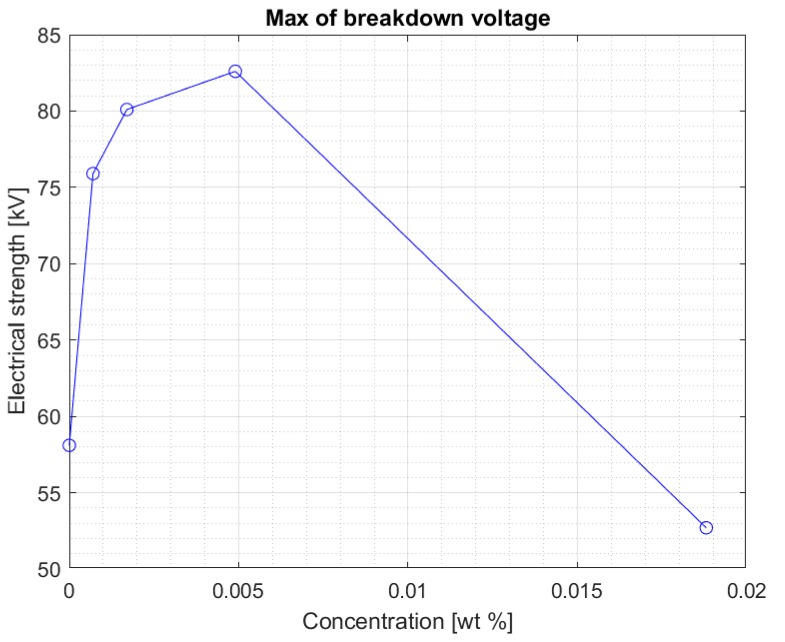
Maximum breakdown voltage values achieved for different nanoparticle concentration in the doped ester after ten weeks mixing.

Figure 8 and Figure 9 show the median and maximum values for measuring the breakdown voltage of the samples after 10 weeks of doping. Once again, an increase in the values can be observed, especially for a 0.0017% sample, but attention is drawn to nanofluids with a 0.0049% nanoparticle in the liquid. Comparing the values for this sample with the breakdown voltage values for pure Midel 1204 ester, a significant increase can be observed, taking into consideration that in previous measurement tests, the values were lower.

**Figure 10:**
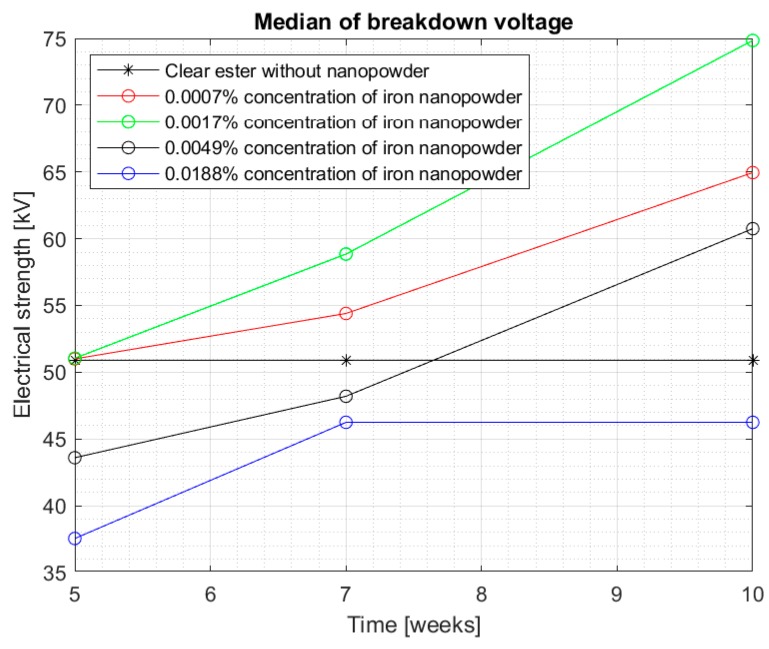
Median of all measurement results for each nanofluid from five to ten weeks mixing.

**Figure 11:**
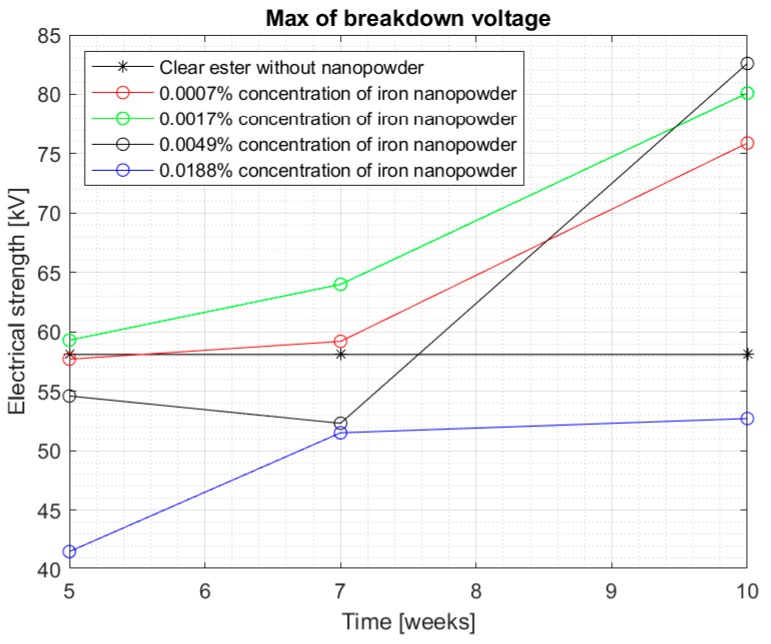
Maximum breakdown voltage values achieved for different nanoparticle concentration in the doped ester from five to ten weeks mixing.

Figure 10 and Figure 11 show all the results obtained during the 10 weeks of the test, where it is easier to see an improvement in electrical strength for each concentration over time. In Figure 10, the median of all the results obtained can be seen, while Figure 11 shows the maximum values obtained for the tested samples. Both figures suggest that the influence of the time of dissolution of a nanoparticle in mineral oil may be significant. The electrical strength of samples with nanoparticle did not achieve satisfactory measurements, even in 5 weeks after doping. The values started to increase significantly after 7 weeks—especially in samples with 0.0007% and 0.0017% concentrations. However, the values of the breakdown voltage for pure Midel 1204 ester, which was maintained under the same conditions, did not change, as can also be seen in the figures. The maximum results obtained for the mentioned nanofluids were 75.9 kV and 80.1 kV, which means 31.5% and 35% increase in their electrical strength. However, the highest increase in strength is visible for the third sample with 0.0049% concentration of iron nanoparticle in hydrophobic carbon sheath, which is 51.3%. Explaining the reason for the dependence of the breakdown voltage on an extended dissolution time may explain the theory of “electron traps”. It assumes that dissolved conductive nanoparticles capture electrons very quickly, which are transformed into heavy negatively charged nanoparticles. This reduces the speed of streamer formation and, consequently, increases the breakdown voltage. Until the admixture is dissolved, the nanofluid’s electrical strength is not positively affected. It should be remembered that iron nanoparticles in the hydrophobic carbon shell are solid material, and those without additional external interference do not dissolve easily in insulating liquids.

The graphs suggest no improvement in the breakdown voltage value for 0.0188% of the nanoparticle in the liquid after more than 7 weeks of mixing, which may suggest that too high of a concentration of the nanoparticle has no positive effect on electrical strength. The probability of saturation of the sample with dissolved nanoparticles may cause the excess admixture to affect the mixture parameters negatively as a contaminant. However, for the remaining concentrations, a significant increase in breakdown voltage can be observed within 10 weeks after admixing and dissolving the nanoparticles.

Obtaining such a high electrical strength for the liquid under study suggests that iron in the carbon shell can, like iron oxides, improve electro-insulating properties. Other parameters such as tan δ or thermal conductivity should also be examined. The influence of the admixture on aging liquids should also be investigated.

## 4. Conclusions

The results presented in the paper indicate that the iron nanoparticle in the hydrophobic carbon shell is able to increase the breakdown voltage of pure natural ester.

- Taking into account the maximum and average results of the obtained breakdown voltage, especially in the last week of measurements, the dissolution time of the nanoparticle can positively influence the electrical strength of Midel 1204.
- Recorded results for different dopant concentrations indicate that there is a limit concentration of the dopant for which the electrical strength of the substance increases.

As the concentration of the admixture increases, the time needed for its complete dissolution increases. At the same time, too high of an admixture concentration may not dissolve completely in Midel 1204 even after 10 weeks. Considering that one of the elements in the admixture was iron, the electric field during the voltage increase between the electrodes could concentrate the still undissolved nanoparticles. On the other hand, these dissolved ones may cause phenomena associated with the theory of “electron traps”. Research on the mixture of insulating esters and iron nanoparticles in a hydrophobic carbon shell will be continued based on the results presented in the article. Other physicochemical parameters of the mixture important for electrical insulation will be examined. Thermal properties of nanofluids presented in the article and their temperature aging ability will also be investigated. The breakdown voltage values presented in the article constitute important information about the electrical strength of the new mixture and are a good starting point for further research.
